# Diagnostic performances of leucine-rich α-2-glycoprotein 1 and stem cell factor for diagnosis and follow-up of colorectal cancer

**DOI:** 10.1186/s43141-021-00116-3

**Published:** 2021-01-25

**Authors:** Manar S. Fouda, Rokaia M. Aljarwani, Khaled Aboul-Enein, Mohamed M. Omran

**Affiliations:** 1 Chemistry Department, Faculty of Science, Helwan University, Ain Helwan, Cairo, 11795 Egypt; 2 Clinical Pathology Department, National Cancer Institute, Cairo University, Giza, Egypt

## Abstract

**Background:**

Colorectal cancer (CRC) is one of the most frequently diagnosed tumors worldwide with high mortality and morbidity. There is an urgent need for biomarkers to improve the outcomes and early detection of CRC. The sensitivity of traditional CRC tumor markers (carcinoembryonic antigen (CEA) and carbohydrate antigen 19-9 (CA19-9)) is not ideal. The levels of leucine-rich-alpha-2-glycoprotein 1 (LRG1) and stem cell factor (SCF) were evaluated, but the combined value of both markers is unclear. This case-control study included four groups: CRC patients before treatments (*n* = 22), CRC patients after treatments (*n =* 26), 20 patients with benign tumor, and 20 healthy subjects. Levels of routine biochemical and hematological markers, traditional tumor markers (CA19.9 and CEA), and candidate markers (LRG1 and SCF) were determined. Univariate and multivariate logistic regression analysis and area receiver-operating characteristic analysis (ROC) were used for evaluation the diagnostic performances of single and combined markers.

**Results:**

No significance difference in traditional tumor markers CEA, CA 19.9, and neutrophil–lymphocyte ratio (NLR) were found among study groups. SCF, LRG1, and platelet–lymphocyte ratio (PLR) were significantly decreased (*p* < 0.05) in non-treated CRC patients than after treated CRC. The combination between SCF and LRG1 showed highly significant difference in CRC patients compared with benign, healthy subjects, and among CRC groups (treated and non-treated) (*p* < 0.0001). The highest areas under curve (AUCs) were observed when LRG1 was used as a single predictor for discriminating CRC from healthy (0.87), benign (0.84), and non-treated CRC vs treated CRC (0.82). AUCs were jumped to 0.90, 0.84, and 0.84 when LRG1 and SCF were combined.

**Conclusion:**

Our study revealed that LRG1 and SCF were potential diagnostic and follow-up markers for CRC.

## Impact statement

Colorectal cancer is a major health problem worldwide. The current work aimed to evaluate the diagnostic performances of CEA, CA19-9, LRG1, and SCF as single and combined markers.

## Background

Worldwide, CRC is the third most commonly detected cancer, and being generally symptomatic, it is often detected in advanced stage of growth [1]. The most reliable method for the CRC screening is endoscope, but it physically burdens and has high cost. Thus, alternative, noninvasive markers have been evaluated and validated to improve early detection and treatment outcome of CRC [2]. In addition, the blood examinations are accessible and easily performed, but the precision to detect the CRC in early stages is restricted. The routine tumor markers (CEA and CA 19–9) are raised in malignancies and benign diseases and have unsatisfactory sensitivity and specificity [3]. LRG is a plasma glycoprotein elevated in the acute-phase response to bacterial or viral infection [4, 5]. LRG1 has several biological functions including cell migration, cell proliferation, cell apoptosis, immune response and neovascularization, and malignancy [5, 6]. Stem cell factor is one of the hematopoietic cytokines (HC) which induce hematopoietic progenitor cell proliferation. Receptor of HC has been established on non-hematopoietic cancer cell lines including CRC [7]. Clinical prospective studies open the door for non-invasive biomarkers which assist in monitoring therapy for CRC patients. CEA levels should be detected before starting in chemotherapy and every 2 to 3 months [8]. In our study, we aimed to evaluate diagnostic performances of traditional CRC tumor markers (CEA, CA19.9) vs LRG1 and SCF for early CRC diagnosis and assess its potential usefulness for monitoring CRC patient’s treatment.

## Methods

### Patients

Sixty-eight patients were recruited from Egyptian National Cancer institute. Patients were divided into four groups: CRC patients before treatments (*n* = 22), CRC patients after treatments (*n =* 26) with histologically affirmed adenocarcinoma of colon or rectum cancer, 20 patients with benign tumor (includes hyperplastic or adenomatous polyps), and 20 healthy subjects. Treatment strategies included chemotherapy, radiotherapy, concurrent chemo and/or radiotherapy, or surgery. Clinical data of patients including age, sex, tumor histology, stage, and treatment strategies were collected from medical records. Pretreatment staging included the following: physical and blood examinations (CEA, CA 19.9, NLR and PLR), computed tomography (CT), chest roentgenogram, abdominal ultrasound scanning, and colonoscopy. TNM (tumor, node, and metastasis) classification system for colorectal cancer was utilized to play out the pathological staging of the study [9].

### Biochemical analyses

Blood samples were assembled from all individuals after clinical determination and blood samples were inspected for routine laboratory examinations, including biochemical profile [liver function tests] and [kidney function tests] using an automated biochemistry analyzer (Beckman Coulter AU680 chemistry analyzer, USA). Complete blood count was estimated using an automated hematology analyzer (Sysmex XN 1000, Japan). Serum CA19.9 and CEA were estimated by ELISA assay according to the manufacturer’s protocol (ARCHITECT i 1000 SR immunoassay analyzer, Abbott USA). LRG1 (Assay Pro/Human LRG1, MO, USA) and SCF Glory Science Co. Ltd/Human SCF/Del Rio, TX, USA) were determined by immunoassay according to the manufacturer’s protocol.

### Statistical analyses

Statistical Package for the Social Sciences (SPSS) operating system, version 22.0 (SPSS Inc., Chicago, IL), was utilized to operate the statistical analysis information. Continuous data was presented as mean ± standard deviation (SD). Statistically significant differences were determined using Kruskal-Wallis, ANOVA, Mann-Whitney *U* test, and Student’s *t* test. Univariate and multivariate logistic regression analysis and AUC were used for evaluation the diagnostic performances of routine and candidate tumor markers. Platelet count-to-lymphocyte ratio (PLR) was calculated as the platelet count divided by the lymphocyte count. Neutrophil-to-lymphocyte ratio (NLR) was calculated as the neutrophil count divided by the lymphocyte count. The best cutoff points and diagnostic performances were determined based on receiver-operating characteristic analysis (ROC). The best combination was developed using the logistic regression function that combined the most discriminatory independent factors. The combination can be represented as = (1.445 + LRG (μg/ml) × 0.038 + SCF (ng/ml) × 0.001).

## Results

### Baseline clinical features of study groups

Baseline clinical features are listed in Table 1; 48 patients (24 men and 24 women) with diagnosis of CRC including 22 non-treated CRC and 26 treated CRC, 20 with benign (13 men and 7 women), and 20 healthy subjects (14 men and 6 women) were enrolled into this study. Levels of routine laboratory parameters show non- significant difference between CRC and benign compared with healthy subjects.

**Table 1:** Clinicopathologic parameters of patients with colorectal cancer and control characteristics

Parameters	Value (%)
**Cancer patients no.**	
**No. of patients**	48
**Treated CRC**	26 (54.2%)
**Non-treated CRC**	22 (45.8%)
**Mean age, years (range)**	48 (28-81)
**Cancer position, no.**	
**Colon**	28 (58.3%)
**Rectal**	20 (41.7%)
**Tumor size**	
**T1**	7 (14.6%)
**T2**	19 (39.6%)
**T3**	10 (20.8%)
**Unknown**	12 (25%)
**Tumor grade, no.**	
**G1**	2 (4.2%)
**G2**	26 (54.2%)
**G3**	9 (18.7%)
**Unknown**	11 (22.9%)
**Lymph node status, no.**	
**Positive**	6 (12.5%)
**Negative**	30 (62.5%)
**Unknown**	12 (25%)
**Metastasis**	
**Positive**	3 (6.25%)
**Negative**	33 (68.75%)
**Unknown**	12 (25%)
**Stage, no.**	
**I**	23 (47.9%)
**II**	7 (14.6%)
**III**	3 (6.25%)
**IV**	3 (6.25%)
**Unknown**	12 (25%)
**Benign cancer diseases**	
**No. of patients**	20
**Mean age, years (range)**	51 (27-73)
**Healthy controls**	
**No. of cases**	20
**Mean age, years**	34 (23-61)

### Levels of biomarkers in study groups

The levels of CEA, CA19.9, PLR, and SCF and LRG1 in benign patients, CRC (treated and non-treated), and healthy subjects are shown in Table 2. The levels of SCF and LRG1 were significantly increased in patients with CRC than in benign (*p* = 0.001) and healthy subjects (*p* = 0.001). No significant difference in traditional markers CEA, CA19.9, and PLR among study groups. SCF, LRG1, and PLR were significantly decreased (*p* values raged from 0.014 to 0.001) in non-treated CRC patients than treated CRC. The combination between SCF and LRG1showed highly significant difference in CRC compared with benign and healthy subjects and among CRC groups (treated and non-treated) (*p* value ranged from 0.001 to < 0.0001).

**Table 2:** Levels of traditional and candidate markers in studied groups

Study groups	CEA (ng/ml)	CA19.9 (μ/ml)	PLR	SCF (ng/ml)	LRG1 (μg/ml)	SCF-LRG1 (μg/ml)
**Healthy subjects**	43.1 ± 14.0	98.1 ± 32.2	4.8 ± 2.2	231.9 ± 33.2	39.6 ± 16.3	3.1 ± 0.64
**Benign**	88.8 ± 21.8	118.7 ± 37.5	5.2 ± 2.3	440.1 ± 35.4	44.6 ± 8.7	3.6 ± 0.38
**CRC**	47.7 ± 13.5	108.5 ± 34.2	5.0 ± 2.2	605.7 ± 38.5	52.5 ± 16.9	4.6 ± 1.3
***p*** **value** ^**a**^	0.349	0.216	**0.025**	0.001	**0.0001**	**0.0001**
**Non-treated CRC**	40.1 ± 33.7	155.9 ± 49.3	6.2 ± 2.1	1185.3 ± 39.5	69.3 ± 12.0	5.3 ± 1.6
**Treated CRC**	26.0 ± 22.2	68.6 ± 48.3	4.2 ± 1.9	530.2 ± 39.1	54.2 ± 13.2	4.0 ± 0.59
***p*** **value**^**b**^	**0.978**	**0.167**	**0.014**	**0.019**	**0.001**	**0.001**

^a^For discriminating among studied groups

^b^For discriminating between non-treated and treated CRC groups

Colon cancer patients had significant high level of SCF compared with rectum cancer (*p* = 0.039) whereas LRG1 levels were significantly elevated in high grade G3 than low grade of CRC (*p* = 0.05). Level of SCF was highly significantly higher in high depth T3 than T2 and T1 (*p* = 0.001), while LRG1 level was significantly higher in T1 than high depth (*p* = 0.032). Levels of CEA, CA19.9, PLR, SCF, and LRG1 had non-significant difference in lymph nodes and presence or absence of metastasis in CRC patients and early or late tumor stage (*p* ≥ 0.05).

### The diagnostic power of single and combined markers

**Fig. 1:**
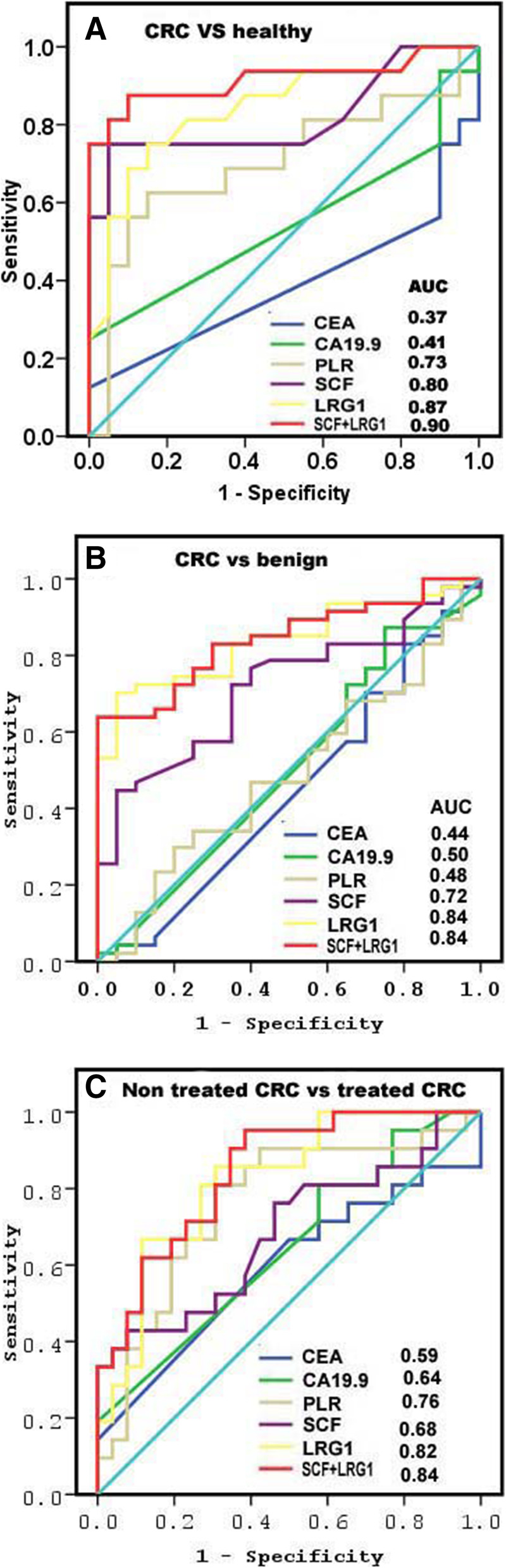
Diagnostic power of single and combined markers. **a** CRC from healthy individuals. **b** CRC from benign. **c** Treated CRC from non-treated CRC patients

ROC analysis was performed to evaluate the discriminatory power of single or combined markers to differentiate patients with CRC from benign and healthy individuals (Table 3). The highest discriminatory AUC was observed when LRG1was used as a single marker with AUC value of 0.87 which jumped to 0.90 when LRG1 and SCF were combined in discriminating CRC from healthy subjects (Fig. 1a). LRG1 recorded the highest efficiency, specificity, positive prediction, and AUC value (66%, 95% and 96%, 0.84, respectively) as an individual marker for discriminating CRC from benign tumors, while AUC was not improved when LRG1 combined with SCF (Fig. 1b). LRG1 had an excellent ability to monitor the treatment (AUC = 0.82) with highest efficiency of 73%. Combined LRG1 and SCF increased AUC to 0.84 for discriminating treated CRC patients from non-treated (Fig. 1c).

**Table 3:** Diagnostic performances of single and combined markers

Variables	AUC	Cut-off	Sensitivity (%)	Specificity (%)	PPV (%)	NPV (%)	Efficiency (%)
**Healthy vs CRC**							
**CEA ng/ml**	0.37	2.8	79	10	68	17	59
**CA 19.9 μ/ml**	0.41	23.4	77	10	67	15	57
**PLR**	0.73	4.9	47	65	76	34	52
**SCF ng/ml**	0.80	539	58	90	93	47%	63
**LRG1 μg/ml**	0.87	60	52	90	93	44	68
**SCF-LRG1**	**0.9**	**3.6**	**85**	**80**	**91**	**70**	**84**
**CRC vs Benign**							
**CEA ng/ml**	0.44	2.8	79	20	70	29	62
**CA 19.9 μ/ml**	0.50	23.4	77	30	73	35	63
**PLR**	0.48	4.9	45	60	72	32	49
**SCF ng/ml**	0.72	539	58	70	82	41	61
**LRG1 μg/ml**	0.84	60	54	95	96	46	66
**SCF-LRG1**	**0.84**	**3.6**	**79**	**70**	**86**	**58**	**77**
**Treated CRC vs non-treated CRC**							
**CEA ng/ml**	0.59	2.8	82	23	47	60	50
**CA 19.9 μ/ml**	0.64	23.4	82	27	49	64	52
**PLR**	0.76	4.55	71	69	65	75	70
**SCF ng/ml**	0.68	539	73	54	57	70	63
**LRG1 μg/ml**	0.82	61.5	82	73	72	83	73
**SCF-LRG1**	**0.84**	**3.6**	**77**	**70**	**68**	**78**	**77**

## Discussion

CRC is one of the main causes of death worldwide, so it is very imperative to early detect CRC. Levels of CEA and CA 19–9 were raised in malignancies and benign diseases and are not specific to malignancies only [10]. The sensitivity and specificity of traditional markers (CEA and CA 19–9) were limited in differentiation malignant from benign disease. We evaluated the diagnostic power of LRG1, SCF, PRL, CEA, and CA 19–9 for early detection of CRC. Our study was the first one that estimated the levels of SCF and LRG1 in CRC patients. Our results demonstrated that SCF and LRG1 levels were significantly increased in patients with CRC compared with benign tumors and healthy subjects. High level of LRG has been attended related to various diseases, such as inflammation and cancer [11, 12]. LRG was informed to show critical roles in CRC tumorigenesis [13] and could be considered as a diagnostic tumor marker [14, 15]. Inflammation response is associated not only to tumorigenicity but also to tumor progression. Inflammatory response plays a dual role in tumorigenicity due to its induced local accumulation of different types of white blood cells that secrete cytokines to stimulate tumor angiogenesis and metastasis, and the rise of monocytes and lymphocytes counts creates a resistance to tumor invasion. Tumor progression is induced by the production of inflammatory cytokines and chemokines by white blood cells, which are themselves stimulated by the tumors [16]. Elevated level of several circulating cytokines was detected in patients with CRC and other malignancies diseases [17, 18]. In the present study, the SCF and LRG1 levels were significantly decreased in treated CRC patients than non-treated CRC patients, while the level of CEA and CA 19–9 had no significant difference in treated CRC compared with non-treated CRC individuals. During treatment of CRC, CEA and CA19-9 traditional tumor markers were undergoing dynamic changes [19, 20]. Our results disagreed with the results of Perez et al. [21]. The levels of CEA were decreased after CRC therapy. Our levels of LRG1 and SCF were correlated with clinicopathological characters and agreed with Kaminska et al. [22]. Increased hematopoietic cytokines correlated with clinicopathological features, and it may have diagnostic value [23].

In the present study, LRG1 has the highest discriminatory power as a single predictor for discriminating CRC from healthy subjects with an AUC value of 0.87, which jumped to 0.90 when SCF and LRG1 were combined. For discriminating patients with CRC from benign, LRG1 has the highest AUC of 0.84 which improved to 0.89 when LRG1 and SCF combined. LRG1 had a great ability to monitor the treatment of CRC with an AUC value = 0.82. Combination of different biomarkers may enhance the diagnostic power and would be used for diagnosis of cancers. The specificity, sensitivity, and AUC of combined CEA and LRG-FT were higher than each marker alone [22]. The AUC of SCF was higher than the IL-3 but less than the AUCs of CEA and CA 19–9 [24]. Mroczko et al. [18] reported that combination of SCF with granulocyte-macrophage colony-stimulating factor (GM-CSF) had greatest diagnostic sensitivity, but GM-CSF and CEA combination had the highest specificity and positive and the negative predictive values. The AUC for osteopontin (OPN), B7-H4, tissue polypeptide-specific antigen, and TPS were 0.81, 0.86, 0.83, and 0.81, respectively. The sensitivity and specificity of B7-H4 for discriminating CRC patients from healthy controls were 88.2% and 86.7%, respectively. However, combination of CEA and B7-H4 had AUC with 0.93, 80% specificity, and 99% sensitivity to differentiate between CRP patients and healthy controls [25]. AUC of miR-125a-3p was 0.69 and 0.84 for CEA for differentiating CRC patients from healthy. Combined CEA and miR-125a-3P enhanced the AUC to 0.86% [26]. AUC of CEA was 0.86 followed by CA 19–9, CYFRA 21-1, IL-8, CA 125, and OPN reaching AUCs between 0.70 and 0.74. AUC of CEA and CA 19–9 increased to 0.89 [27]. This study had two limits. First, it included a small population size and the second limit was including cancer rectum with colon cancer. In the coming research papers, we will increase the population size and include cancer rectum as a separate disease group.

## Conclusions

LRG1 had the highest AUC for discriminating patients with CRC from healthy and benign individuals and treated CRC from non-treated CRC. LRG1 combined with SCF could be considered a promising candidate marker for CRC diagnosis and follow-up.

## Data Availability

The authors declare that all generated and analyzed data are included in the article.

## Abbreviations

AUC: Area under the curve
CA19-9: Carbohydrate antigen 19-9
CEA: Carcinoembryonic antigen
CT: Computed tomography
CRC: Colorectal cancer
HC: Hematopoietic cytokines
GM-CSF: Granulocyte-macrophage colony-stimulating factor
LRG1: Leucine-rich-alpha-2-glycoprotein 1
OPN: Osteopontin
NLR: Neutrophil–lymphocyte ratio
NPV: Negative predictive value
PPV: Positive predictive value
PLR: Platelet–lymphocyte ratio
ROC: Receiver-operating characteristic analysis
SCF: Stem cell factor
SD: Standard deviation
SPSS: Statistical Package for the Social Sciences
TNM: Tumor, node, and metastasis
